# Trends in food and nutrition behaviours, knowledge and attitudes among youth in six countries: findings from the 2019–2021 International Food Policy Study Youth Surveys

**DOI:** 10.1017/S1368980024002416

**Published:** 2024-11-27

**Authors:** Rachel B Acton, Christine M White, Karen Hock, Lana Vanderlee, David Hammond

**Affiliations:** 1 School of Public Health Sciences, University of Waterloo, Waterloo, ON N2L3G1, Canada; 2 École de Nutrition, Centre nutrition, santé et société (Centre NUTRISS) and Institut sur la nutrition et les aliments fonctionnels (INAF), Université Laval, Québec, Canada

**Keywords:** Nutrition policy, Adolescent, Child, Policy analysis, Food labelling, Food security

## Abstract

**Objective:**

This commentary highlights the release of findings now available in the report *International Food Policy Study Youth Surveys: Summary of Findings 2019–2021.*

**Design:**

The survey data described in this commentary consist of repeated cross-sectional surveys conducted annually beginning in 2019.

**Setting:**

Online surveys were conducted in 2019 to 2021 among respondents living in Australia, Canada, Chile, Mexico, the United Kingdom and the USA.

**Participants:**

Survey respondents were youth aged 10–17 years in 2019 (*n* 12 031), 2020 (*n* 11 108) and 2021 (*n* 10 459).

**Results:**

The report described in this commentary summarises findings on food and nutrition behaviours, attitudes and knowledge among youth, including their diet sources and patterns, school nutrition environments, food security, diet intentions, weight perceptions and weight loss behaviours, sugary drink perceptions, awareness of public education and mass media campaigns, perceptions of food labels and exposure to food and beverage marketing.

**Conclusion:**

Results from the IFPS Youth surveys provide important insights into key policies of global interest, including front-of-package nutrition labelling, levies on sugary beverages and restrictions on marketing unhealthy food and beverages to children. As policymakers continue to seek effective strategies to improve adolescent health outcomes, ongoing cross-country monitoring of food and nutrition-related indicators, such as the data from the International Food Policy Study, will be critical in assessing dietary trends and evaluating upcoming policies.

Poor dietary intake remains one of the leading causes of non-communicable disease globally^(1)^. Given that dietary behaviours learned in childhood often persist into adulthood, adolescents are an important population for enhancing dietary intake^(1)^. Population-level strategies such as front-of-package nutrition labelling, levies on sugary beverages and restrictions on marketing unhealthy food and beverages to children are increasingly being used to shape food environments and support healthy eating^(2)^. There are notable challenges to evaluating national-level dietary policies, including the difficulty of assessing their impacts against the variable and complex backdrop of a country’s food environment, the time required to evaluate long-term outcomes of dietary changes and the fact that policies are often implemented with little advanced notice. Therefore, there is relatively little ‘real world’ or ‘post-implementation’ data available to assess their impacts on adolescent health. To address this gap, the International Food Policy Study (IFPS) was initiated to establish a ‘natural experiment’ research design by collecting data annually on individuals’ knowledge, attitudes and behaviours related to food and nutrition across multiple countries with different policy environments. This commentary highlights the release of findings from the 2019 to 2021 IFPS Youth Surveys, now available in the report *International Food Policy Study Youth Surveys: Summary of Findings 2019–2021*^(3)^.

The IFPS Youth surveys consist of repeated cross-sectional surveys, which have been conducted annually since 2019 among youth aged 10–17 years in six high- and upper-middle-income countries (Australia, Canada, Chile, Mexico, the UK, and the USA. The IFPS Youth surveys are part of a larger study that surveys adults in the same countries, excluding Chile. The surveys are collected online through the Nielsen Consumer Insights Global Panel and their partners’ panels. More details on the sampling and data collection methods are available in the full report^(3)^ and in the IFPS Youth Technical Reports available at www.foodpolicystudy.com/methods.

The full report presents findings from the 2021 IFPS Youth surveys (*n* 10 459), as well as results for selected outcomes from 2019 (*n* 12 031) and 2020 (*n* 11 108). The report summarises findings on a range of topics pertaining to food behaviours, attitudes and knowledge among youth, including their diet sources and patterns, school nutrition environments, food security, diet intentions, weight perceptions and weight loss behaviours, sugary drink perceptions, awareness of public education and mass media campaigns, perceptions of food labels and exposure to food and beverage marketing.

Results from the IFPS Youth surveys provide important insights into key policies of global interest, such as front-of-package nutrition labels. For example, youth in Chile and Mexico – where ‘high in’ warning-style front-of-package nutrition labels are mandatory on products with excess nutrients of concern^(2)^ – consistently reported greater awareness, use and understanding of their country’s front-of-package warning labels compared to those in Australia and the UK, where voluntary Health Star Rating and traffic light labels are used,^(2)^ and compared to the ‘guideline daily amount’ labels previously used in Mexico^(4)^. These results generally mirrored trends observed among adults^(5)^. Although national front-of-package labelling systems are not currently required in Canada or the USA, ‘high in’ labels for foods with excess sugars, sodium and saturated fats will be required by January 2026 in Canada^(6)^, and the USA is studying options for a national front-of-package label design^(7)^. The IFPS surveys will be well positioned to capture the impacts of the these new front-of-package policies once implemented.

Notably, the surveys also revealed that youth’s food sources and behaviours reflect the growing presence of digital media in their day-to-day lives: the majority of youth in all six countries reported ordering food online using an app, with about one-third to one-half of respondents in each country reporting ordering in the past month. When asked about their use of digital media – a prominent source of food and beverage marketing – over half of youth in most countries reported spending more than 1 h per day watching television or movies, watching YouTube and playing games on smartphones, computers or game consoles.

The IFPS Youth surveys also monitor trends in food security. The proportion of youth experiencing any food insecurity was relatively stable between 2019 and 2021 for most countries, with some exceptions. In Mexico, the total proportion of food insecure youth increased between 2019 and 2020, followed by slight reductions in 2021. These changes may have been a result of the COVID-19 pandemic financial impacts in 2020. In contrast, there were notable increases in the proportion of youth reporting ‘several’ or ‘many’ food insecurity experiences in Canada and the UK between 2020 and 2021 to levels above those observed in 2019, possibly due to the removal of COVID-19 pandemic-related income supports available in these countries throughout 2020^(8,9)^.

Results from the surveys also revealed notable COVID-19 pandemic-related impacts on youth’s access to food at school, which is an important food environment for adolescents^(10)^. There was a clear and substantial drop in 2020 in the proportion of youth in both Chile and the USA – countries where nearly all schools provide free school meals^(11)^ – who reported getting food from free lunch programs. In the USA, youth’s reported use of free lunch programmes dropped from 56 % in 2019 to 21 % in 2020, while in Chile these rates dropped from 46 % in 2019 to only 2 % in 2020. These drastic changes in access to food at school were likely to have had substantial impacts on families’ abilities to provide adequate foods for their children.

As policymakers continue to seek effective strategies to improve adolescent health outcomes, ongoing cross-country monitoring of food and nutrition-related indicators will be critical in assessing dietary trends and evaluating upcoming policies^(6)^.
